# Acoustic effects of medical, cloth, and transparent face masks on speech signalsa)

**DOI:** 10.1121/10.0002279

**Published:** 2020-10-27

**Authors:** Ryan M. Corey, Uriah Jones, Andrew C. Singer

**Affiliations:** Coordinated Science Laboratory, University of Illinois at Urbana-Champaign, Urbana, Illinois 61801, USA

## Abstract

Face masks muffle speech and make communication more difficult, especially for people with hearing loss. This study examines the acoustic attenuation caused by different face masks, including medical, cloth, and transparent masks, using a head-shaped loudspeaker and a live human talker. The results suggest that all masks attenuate frequencies above 1 kHz, that attenuation is greatest in front of the talker, and that there is substantial variation between mask types, especially cloth masks with different materials and weaves. Transparent masks have poor acoustic performance compared to both medical and cloth masks. Most masks have little effect on lapel microphones, suggesting that existing sound reinforcement and assistive listening systems may be effective for verbal communication with masks.

## I. INTRODUCTION

As the world works to control the novel coronavirus 2019 (COVID-19) pandemic, face masks are expected to prove critical to slowing the spread of the virus. However, it can be difficult to understand speech when the talker is wearing a mask, especially for listeners with hearing loss (Chodosh *et al.*, 2020; Tucci, 2020). By studying the acoustic effects of masks on speech signals, we can learn which masks are best for speech transmission and find ways to make communication easier.

Most prior research on masked speech has focused on medical equipment such as surgical masks and N95 respirators. Recent acoustic studies have shown that surgical masks and N95 respirators can attenuate higher-frequency sounds by between 3 and 12 dB (Atcherson *et al.*, 2020; Goldin *et al.*, 2020; Wolfe *et al.*, 2020). Listening tests using audio-only recordings made with medical masks have not shown significant effects on speech intelligibility (Mendel *et al.*, 2008; Palmiero *et al.*, 2016; Thomas *et al.*, 2011).

To conserve supplies of medical masks, health authorities have recommended cloth masks, which can be made from household materials or purchased commercially. Recent studies suggest that the efficacy of cloth masks at blocking respiratory droplets depends on the fabric material, weave, and number of layers. Konda *et al.* (2020) found filtration efficiency to be higher for densely woven cotton and hybrid fabrics than for loosely woven cotton. Masks with more layers were more efficient. Aydin *et al.* (2020) found that single-layer masks made of t-shirt fabric were among the most porous materials studied, but three-layer masks performed as well as medical masks. They also found an inverse relationship between breathability and droplet-blocking efficiency.

Because both medical and cloth face masks obstruct visual cues that contribute to speech intelligibility (Llamas *et al.*, 2008), there has been growing interest in transparent face coverings such as plastic shields and face masks with windows (Atcherson *et al.*, 2020; Wolfe *et al.*, 2020). In listening tests with audiovisual recordings of talkers, transparent masks improved intelligibility for listeners with severe-to-profound hearing loss compared to opaque paper masks (Atcherson *et al.*, 2017).

**FIG. 1.**
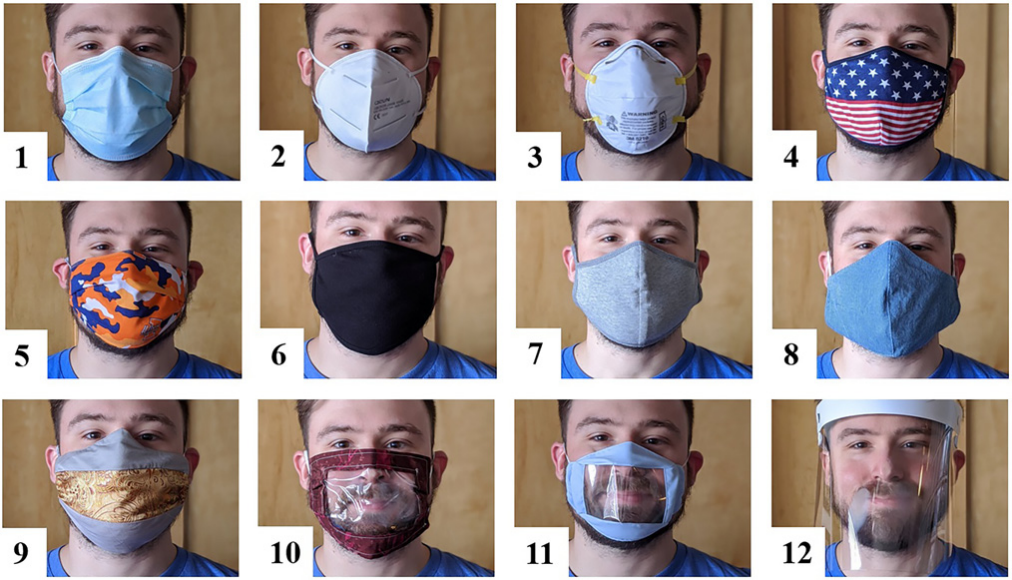
(Color online) Masks used in experiments and described in Table I.

To understand the effects of masks on speech, we measured the acoustic attenuation of a surgical mask, N95 and KN95 respirators, six cloth masks made from different fabrics, two cloth masks with transparent windows, and a plastic shield, as shown in Fig. 1. The measurements used both a head-shaped loudspeaker and a live human talker. The experiments show that different masks have different high-frequency effects and that they alter the directivity of speech. Finally, to examine the effects of masks on sound reinforcement and assistive listening systems, we tested microphones placed on the lapel, cheek, forehead, and next to the mouth. These amplification technologies may prove critical to verbal communication during the pandemic.

## II. METHODS

**FIG. 2.**
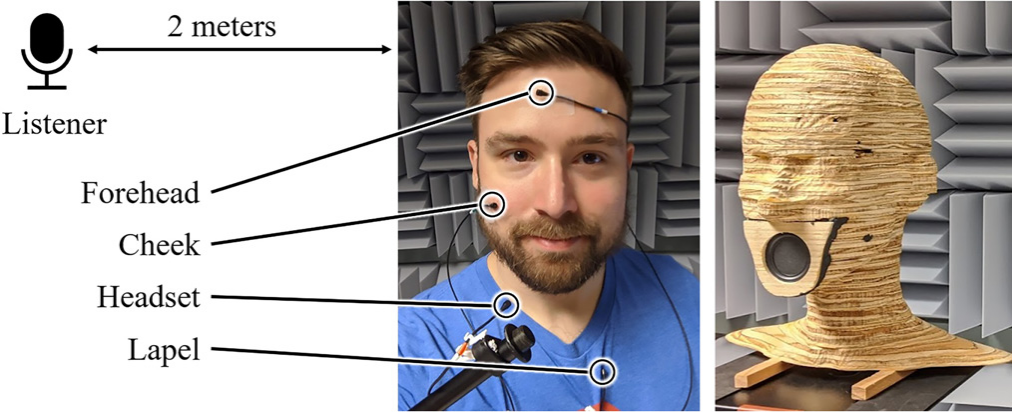
(Color online) Speech signals were produced by a human talker and loudspeaker model. Microphones were placed at listener distance and at several points on and near the face.

To simulate sound heard by a conversation partner, a side-address cardioid condenser microphone (Rode NT1-A) was placed two meters from the talker position. To study the effect of masks on sound reinforcement and assistive listening systems, omnidirectional lavalier condenser microphones (Countryman B3) were placed next to the mouth (“headset” position), on the lapel, on the cheek, and the forehead of the talker, as shown in Fig. 2. All microphones have flat frequency responses from 20 Hz to 20 kHz. The laboratory walls are acoustically treated with 8-in. melamine and 2-in. polyurethane foam wedges.

Sound was produced by two sources. A custom-built head-shaped loudspeaker produced ten-second logarithmic frequency sweeps to measure acoustic transfer functions between the talker and listener positions. The plywood loudspeaker uses a 2-in. full-range driver (Tectonic TEBM35C10-4) and has a directivity pattern that is closer to that of a human talker compared to studio monitors. It has a nominally flat on-axis frequency response over the tested range from 100 Hz to 16 kHz. To characterize the directional effects of masks, the loudspeaker was placed on a turntable and rotated in 15 degree increments while the “listener” microphone remained fixed.

For more realistic speech signals, 30-s read-speech recordings were made from a human talker, who attempted to use a consistent speech level for each recording. Recordings of the human talker were repeated three times non-consecutively with each mask. Human subject research was approved by the University of Illinois Institutional Review Board.

For both the loudspeaker and human experiments, measurements were first taken with no face covering to establish a baseline. The recordings were then repeated with the 12 face coverings listed in Table I and shown in Fig. 1.

**TABLE I.** Mask measurements and 2–16 kHz acoustic attenuation results (mean ± standard deviation).

	Material	Layers	Thickness (mm)	Mass (g)	Speaker atten. at listener (dB)	Human atten. at listener (dB)	Human atten. at lapel (dB)
1	Polypropylene (YY/T 0969)	3	0.4	3	3.6	2.8 ± 1.3	1.0 ± 1.4
2	KN95 respirator (GB 2626)	2	0.6	4	4.0	2.6 ± 1.1	0.0 ± 1.3
3	N95 respirator (3 M 8210)	1	1.5	9	5.7	5.4 ± 1.2	3.6 ± 1.3
4	Cotton jersey (generic)	2	0.7	11	4.0	3.1 ± 1.1	0.5 ± 1.1
5	Cotton plain (handmade)	2	0.5	11	4.0	4.3 ± 1.1	1.4 ± 1.2
6	Cotton/spandex jersey (generic)	3	1.5	16	6.1	5.2 ± 1.5	2.3 ± 1.3
7	Cotton/spandex jersey (LASC)	2	0.9	17	8.2	6.1 ± 1.2	2.0 ± 1.1
8	Cotton plain and denim (Jo-Ann)	2	1.1	21	9.4	10.0 ± 1.3	3.2 ± 1.1
9	Cotton percale bedsheet and polyester trim (handmade)	2	1.0	14	12.6	9.5 ± 1.7	3.1 ± 1.3
10	Cloth and vinyl window (handmade)	1	0.4	12	10.8	7.8 ± 1.2	--2.0 ± 1.5
11	Cloth and PVC window (UTSDesignStore)	1	0.3	7	12.5	8.0 ± 1.7	0.4 ± 1.6
12	Plastic shield (generic)	1	0.4	50	13.7	8.2 ± 1.2	--7.6 ± 1.3

## III. RESULTS AND DISCUSSION

### A. Acoustic attenuation of face coverings

**FIG. 3.**
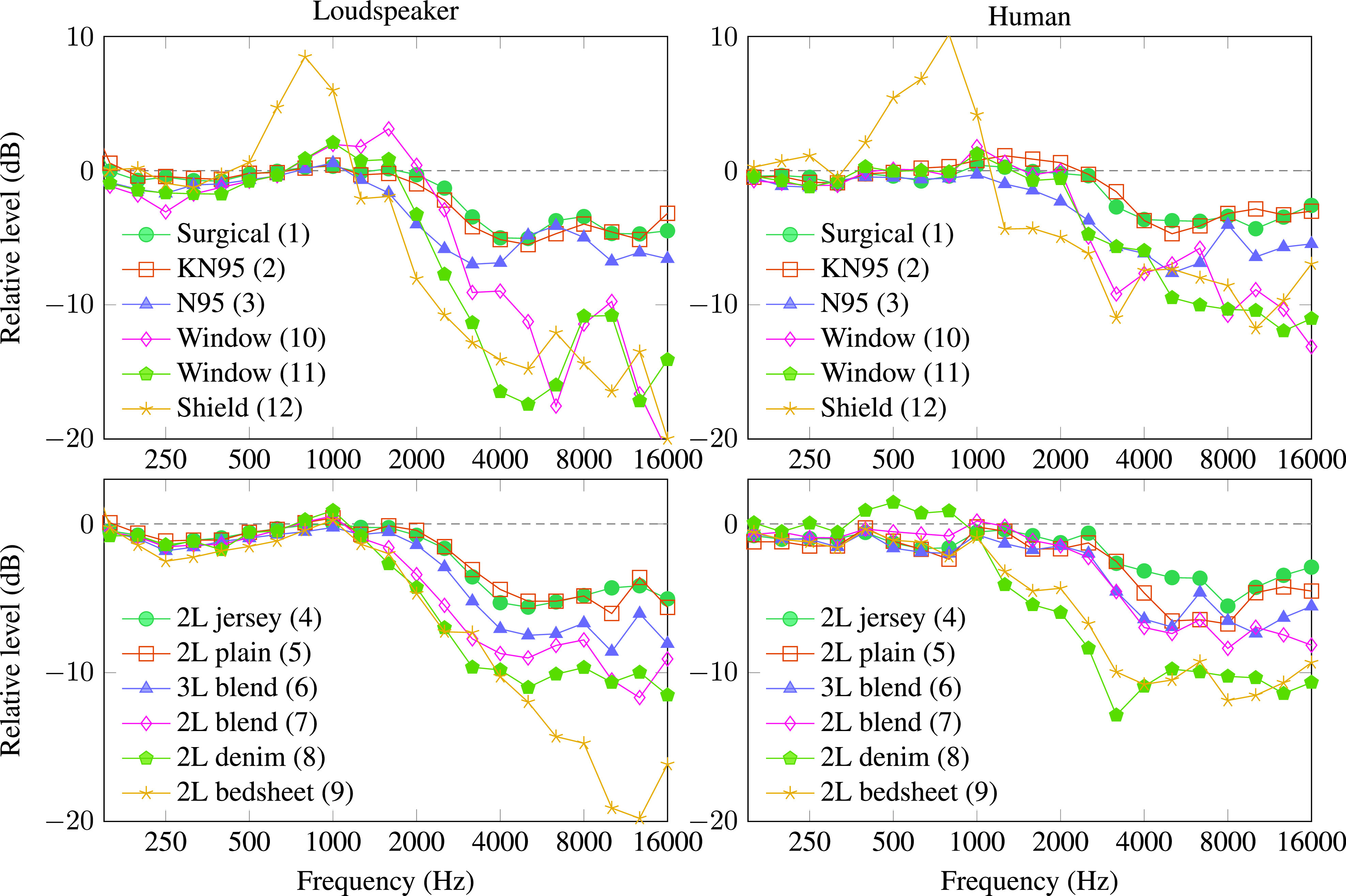
(Color online) Effect of different masks on sound levels measured at the listener position for a head-shaped loudspeaker (left) and human talker (right). Human speech attenuation values are means over three recordings. The overall standard deviation between human recordings was between about 1 and 2 dB at the plotted frequencies.

Figure 3 shows the effects of several masks measured at the listener position. The plots on the left show the differences in acoustic transfer functions measured with and without masks on the loudspeaker model. The plots on the right show the corresponding results for the human talker averaged over three non-consecutive recordings; the human spectra varied by 1–2 dB between recordings, with larger variation at higher frequencies. The attenuation values shown in Table I are logarithmically weighted averages from 2 to 16 kHz, that is, means of the points shown in the plots.

Most masks had little effect below 1 kHz but they attenuated higher frequencies by different amounts. The surgical mask (1) and KN95 respirator (2) had peak attenuation of around 4 dB, which is consistent with the results reported by Goldin *et al.* (2020) with a head-and-torso simulator. The N95 respirator (3) attenuated high frequencies by about 6 dB, which is similar to the average attenuation reported by Goldin *et al.* (2020).

The cloth masks varied widely depending on material and weave. The 100% cotton masks in jersey (4) and plain (5) weaves had the best acoustic performance and were comparable to the surgical mask. The cotton/spandex blends performed worse. Surprisingly, the 2-layer cotton/spandex mask (7) produced greater attenuation than the 3-layer cotton/spandex mask (6), perhaps because it has a higher proportion of spandex and fit more snugly on the face. Masks made from tightly woven denim (8) and bedsheets (9) performed worst acoustically. It appears that material and weave are the most important variables for acoustic performance: More breathable fabrics transmit more sound.

Finally, the transparent masks (10–12) performed poorly acoustically at high frequencies, blocking around 8 dB for the human talker and 10–14 dB for the loudspeaker. Although these masks are often recommended to help listeners with hearing loss because they preserve visual cues, they also harm the high-frequency sound cues that are crucial for speech.

### B. Effect of face coverings on speech directivity

**FIG. 4.**
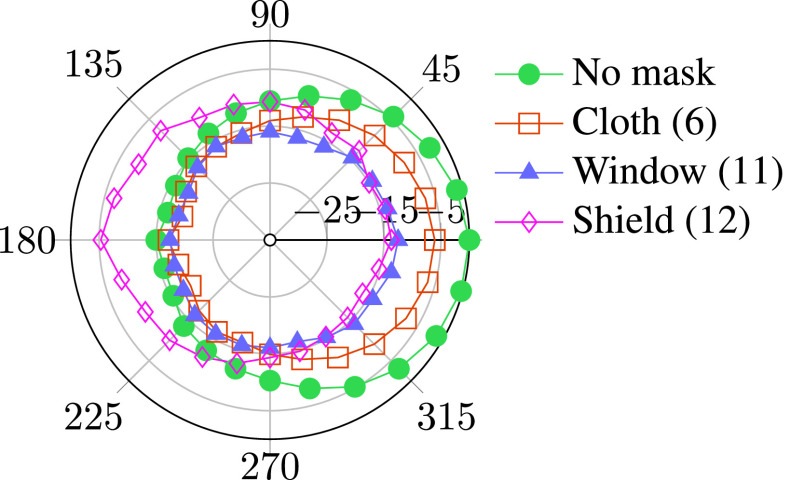
(Color online) Spatial distribution of 2–16 kHz sound energy for a head-shaped loudspeaker with different masks in dB relative to no mask at 0 degrees.

Figure 4 shows the relative high-frequency sound level as a function of angle for the head-shaped loudspeaker. The plot shows a logarithmically weighted average of relative sound level from 2 to 16 kHz. For all masks tested, acoustic attenuation was strongest in the front. Sound transmission to the side of and behind the talker was less strongly affected by the masks, and the shield (12) amplified sound behind the talker. These results suggest that masks may deflect sound energy to the sides rather than absorb it. Therefore, it may be possible to use microphones placed to the side of the mask for sound reinforcement.

### C. Effect of microphone placement

**FIG. 5.**
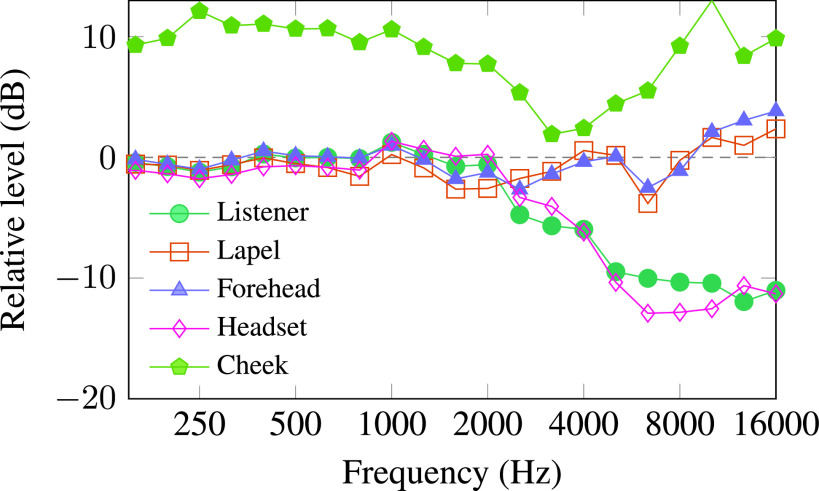
(Color online) Effect of the PVC window mask (11) on sound levels measured at different microphones relative to the same measurements with no mask on a human talker.

Masks attenuate high-frequency sound for distant listeners, but they have different effects on microphones on and near the face. Figure 5 shows the acoustic effects of the polyvinyl chloride (PVC) window mask (11) on different microphones on a human talker. The listener and headset microphones experience similar high-frequency attenuation. The cheek microphone taped under the mask recorded higher sound levels, but with spectral distortion. The lapel and forehead microphones showed small and mostly uniform attenuation over the range of speech frequencies. Similar results were obtained for masks 1–10, although the performance of the cheek microphone varied depending on the shape of the mask. The shield (12) strongly distorted speech spectra for all microphones.

**FIG. 6.**
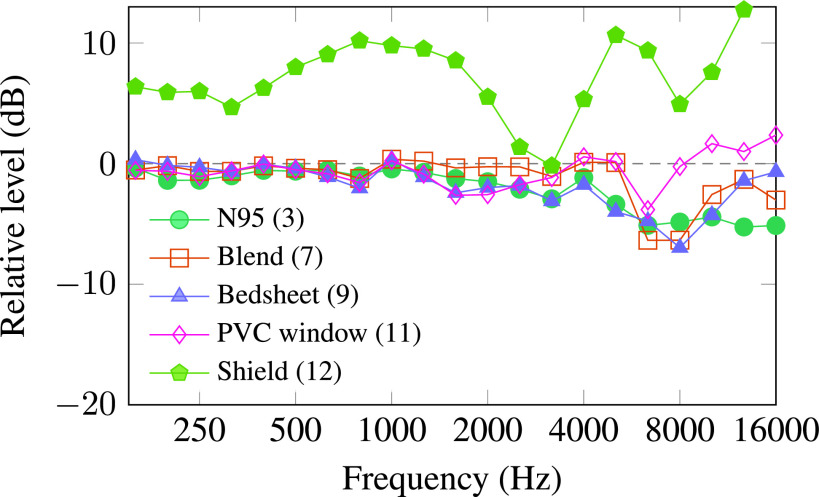
(Color online) Effect of several masks on sound levels at a lapel microphone on a human talker relative to sound levels at the same microphone with no mask.

Figure 6 compares several masks using a lapel microphone. Only the shield has a strong effect on the speech spectrum at the lapel. Sound capture and reinforcement systems used in classrooms and lecture halls often rely on lapel microphones, and remote microphones that transmit to hearing aids are often worn near the chest. These systems should work with masks with little modification. A recent audio-only listening experiment with normal-hearing adults showed that remote microphones can improve intelligibility with some transparent masks (Rudge *et al.*, 2020).

## IV. CONCLUSIONS

These experiments show that face masks attenuate high-frequency sound in front of the talker, with the strongest attenuation above 4 kHz. Surgical masks offer the best acoustic performance among all masks tested. If those masks are not available, loosely woven 100% cotton masks perform well acoustically, although they may offer less protection against small droplets than medical masks. Tightly woven cotton and blended fabrics are less porous but also transmit less sound. Multilayer masks made of loosely woven cotton may offer a reasonable compromise between droplet-blocking efficiency and acoustic performance.

Shields and masks with windows perform much worse acoustically than opaque cloth masks. Fortunately, window masks do not strongly affect the lapel microphones used in sound reinforcement and assistive listening systems. To preserve visual cues without destroying high-frequency sound cues, talkers can wear clear window masks and lapel microphones. Although face masks make verbal communication more difficult, amplification technologies can help people with and without hearing loss to hear each other during the pandemic.
